# The Current Landscape of Repurposed Drugs for Fungal Neglected Tropical Diseases

**DOI:** 10.1007/s12281-025-00504-z

**Published:** 2025-05-29

**Authors:** Tahsin Farid, Keyla C. Tumas, Heather A. Stone, Mili Duggal

**Affiliations:** 1 Office of Medical Policy, Center for Drug Evaluation and Research (CDER), United States Food and Drug Administration, Silver Spring, MD, USA; 2 Division of Preclinical Innovation, National Center for Advancing Translational Sciences (NCATS), National Institutes of Health, Rockville, MD, USA

**Keywords:** Chromoblastomycosis, Sporotrichosis, Eumycetoma, Implantation mycoses, Drug repurposing

## Abstract

**Purpose of Review:**

Eumycetoma, chromoblastomycosis, and sporotrichosis are three of only four fungal infections recognized as Neglected Tropical Diseases (NTDs) by the World Health Organization. They are a significant source of morbidity in subtropical and tropical regions of the Americas, Africa, and Asia. There are very few treatments approved for these diseases. Clinicians often use drug repurposing, off-label use of existing drugs, for their treatment. This article is a systematic review of the published literature on the treatment of fungal NTDs from the last five years (2019–2024). It will provide an overview for each fungal NTD, their current treatment landscape, and the challenges associated with their treatment.

**Recent Findings:**

Itraconazole remains the most widely used antifungal for the treatment of these fungal NTDs. Newer antifungals such as fosravuconazole have matched the efficacy of currently available drugs while reducing adverse events and pill burden. Other promising treatment strategies involve the use of immunomodulators (e.g., imiquimod), steroids (e.g., prednisolone), or non-steroidal anti-inflammatory agents in combination with traditional antifungal agents.

**Summary:**

Frequently repurposed drugs include itraconazole, posaconazole, voriconazole, amphotericin B, terbinafine, potassium iodide, and 5-flucytosine. Most of these drugs have significant side effects, unsatisfactory cure rates, and significant cost that restricts their use. Systematic collection of this drug repurposing data and analyzing it in aggregate using platforms such as CURE ID has the potential to generate efficacy signals for drugs. These promising candidates can then be studied comprehensively in clinical trials for drug approval.

## Introduction

The World Health Organization (WHO) has classified eumycetoma, chromoblastomycosis, and sporotrichosis as neglected tropical diseases (NTDs) [1–3]. All three of these fungal NTDs are primarily caused by environmental fungi predominantly found in subtropical and tropical regions of the Americas, Africa, and Asia. They disproportionately impact socially and economically marginalized communities, who often have limited access to healthcare [1]. Transmission frequently occurs through breaks in the skin from thorns or handling infected materials during activities such as farming and can cause various clinical manifestations [1, 4, 5]. The infections begin as local skin lesion(s) that can disseminate to the skin, fascia, bone, and subcutaneous tissues. This can result in disabilities and contribute to social stigma [1, 5–10].

Existing treatment options are either ineffective or have significant side effects that restrict their use and very few of them are approved by the United States Food and Drug Administration (FDA) for the treatment of these infections [11]. There is an urgent need to develop new medications to meet the clinical requirements for the management of fungal NTDs [12]. These diseases primarily occur in resource-constrained settings, and pharmaceutical companies are not developing new drugs to treat them, as potential profits are unlikely to compensate for the cost of de novo drug discovery [12]. As a result, clinicians treating patients with fungal NTDs often use available drugs that have been approved for other diseases (also known as drug repurposing). Repurposing is an approach that could aid in finding treatments for fungal NTDs, while cutting expenses and shortening the drug discovery process by relying on existing safety and pharmacokinetic profiles of drugs that are already on the market [12].

This article will provide an overview of the three fungal NTDs, the current treatment landscape, and the challenges associated with their treatment. It will review the repurposed drugs used for treatment of fungal NTDs, demonstrate their continued limitations, and describe new advancements in treatments.

## Methodology

### Search Strategy

**Fig. 1:**
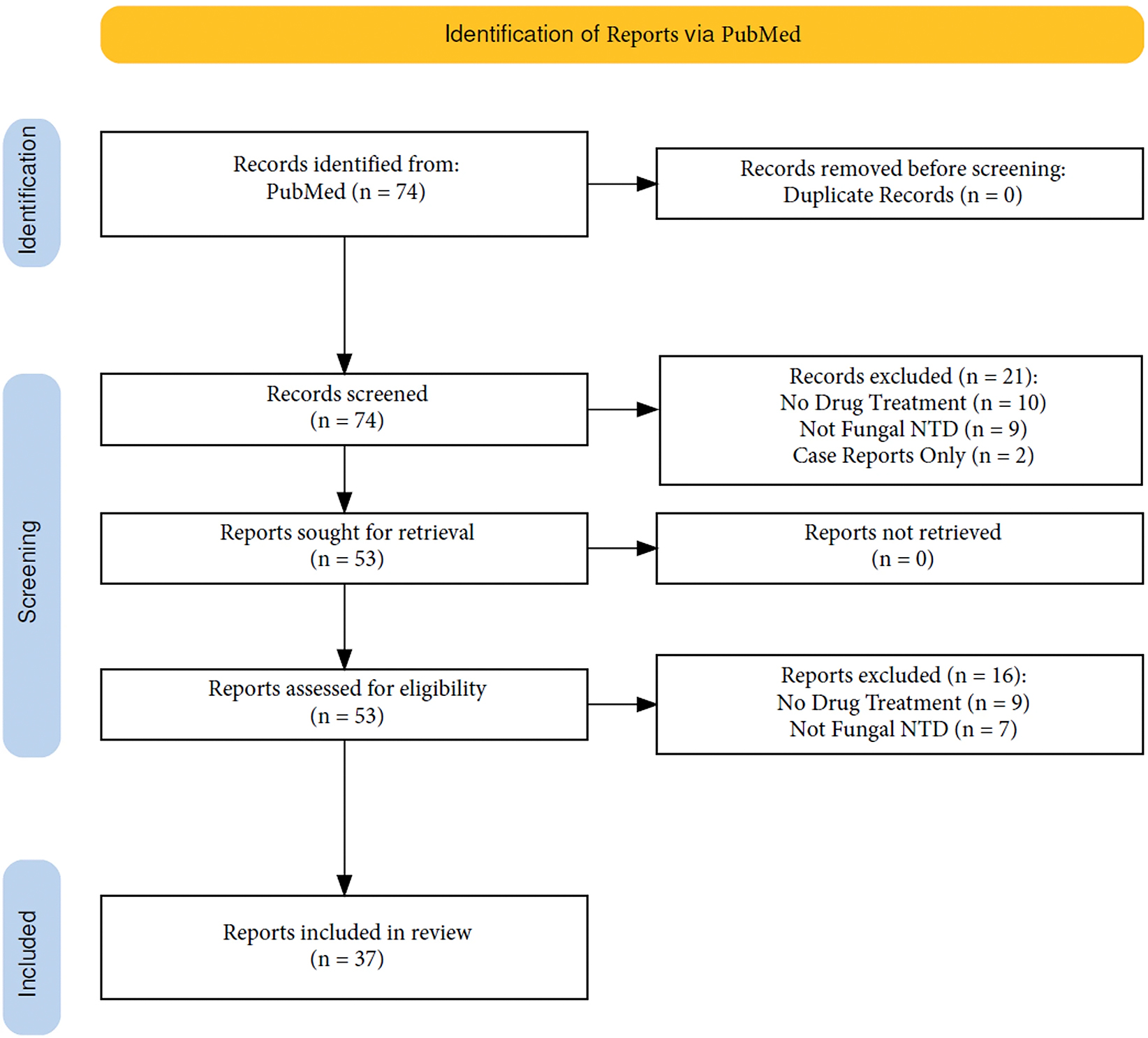
PRISMA flow diagram adopted from Haddaway et al. 2022 [14]

This article is a systematic review of the published literature aimed at identifying all articles from the last five years (2019–2024) reporting treatment information on any fungal NTD (chromoblastomycosis, mycetoma, or sporotrichosis). PubMed was used to retrieve 74 total articles on Fungal NTDs. All identified clinical trials, observational studies, and reviews were included. Individual case reports or case series were excluded; however, if the articles contained a literature review, they were also included. Articles were excluded if they were published before 2019, did not involve human subjects, or were not written in English and did not have an official English translation available. All articles were uploaded to Rayyan.ai and screened according to the PRISMA protocol (Fig. 1) [13, 14].

### Report Selection

From the PubMed search string, 74 articles were identified. Twenty-one of these articles were excluded through screening of titles and abstracts. Ten of the excluded articles did not contain information on fungal NTDs, nine articles did not have drug treatment information, and the remaining two articles were case reports without accompanying literature reviews. On full-text review, 16 articles were excluded, including for not having drug treatment information (*n* = 9) or for not having information on fungal NTDs (*n* = 7). Thirty-seven articles were ultimately included in the systematic review and all relevant information was extracted (see Fig. 1) [14].

## Results

### Chromoblastomycosis

#### Background

Chromoblastomycosis is a chronic fungal infection of the skin and subcutaneous tissues caused by dematiaceous fungi [15]. Australia, India, Brazil, Venezuela, Mexico, Madagascar, and China have reported the highest number of chromoblastomycosis cases [16]. The main causative agents are *Fonsecaea pedrosoi (F. pedrosoi)*, *Fonsecaea monophora (F. monophora)*, *Cladophialophora carrionii (C. carrionii)*, *Exophiala dermatitis (E. dermatitis)*. Among these pathogens, *F. pedrosoi* is the most common species and is primarily found in tropical and subtropical regions around the world [8]. Chromoblastomycosis affects both immunocompetent and immunosuppressed hosts [17]. The duration of incubation remains unclear, and the progression of the disease is gradual and continuous [18]. In most cases, the lower limbs are affected, but infections may occur in the upper limbs and other sites [8]. Initially, the infection typically appears as red papules at the location of exposure. As time passes, these lesions can advance, encompassing broader regions of the skin with nodular, verrucous, tumoral, plaque, or scar-like appearances [18]. The accurate diagnosis of chromoblastomycosis relies on clinical and pathological information and may be confirmed by microbiologic evidence of characteristic thick-walled, single, or multicellular clusters of pigmented fungal cells (also known as medlar bodies, muriform cells, or sclerotic bodies) [17]. The diverse clinical manifestations and causative factors make treatment of this disease challenging [15]. There are no established treatment protocols for chromoblastomycosis [18]. The patient’s treatment depends on the specific pathogen, type of lesion, and the outcome of antifungal susceptibility testing [15].

#### Treatment Landscape

Treatment options for chromoblastomycosis include oral antifungals, surgery, and cryotherapy [19]. Physical approaches such as cryotherapy have limitations such as high relapse indexes and frequently results in discolored and unattractive scars [8]. For early lesions, surgical removal is the preferred initial approach, followed by medication [16]. Relapses are common and can cause severe disability [19].

The preferred antifungals are azoles, with itraconazole (200–400 mg/day) being the most frequently used [16, 20]. Although itraconazole has therapeutic benefits, it is frequently combined with other drugs for improved efficacy [8, 16]. For example, a combination of 5-flucytosine and itraconazole has been used to treat chromoblastomycosis [21]. Terbinafine (500 mg/day), an allylamine, is also commonly combined with itraconazole (200–400 mg/day) to hasten the treatment of chromoblastomycosis [22–25]. Nevertheless, the success rate of this combination varies from 15 to 80% depending on the disease severity and the type of organism [8]. More recently, posaconazole (400–800 mg/day) and voriconazole (200–400 mg/day), both part of the triazole antifungal drug class, have been utilized to treat chromoblastomycosis. Both medications were successfully used in patients with chronic chromoblastomycosis lesions that did not respond to conventional treatments [26, 27].

Small studies have been carried out on patients with chromoblastomycosis to demonstrate that combining traditional antifungal drugs with immunomodulatory agents may help in situations where patients don’t respond to conventional treatments [8]. Imiquimod is an immunomodulator that has recently been added to chromoblastomycosis combination therapy. This medication is authorized by both the European Medicine Agency (EMA) and the FDA to treat actinic keratosis, human papillomavirus-caused genital warts, and superficial basal carcinoma. Research indicated that imiquimod alone, or in combination with itraconazole or itraconazole plus terbinafine notably improved the lesions in four patients suffering from chromoblastomycosis due to *F. pedrosi* [28]. Another study revealed that three patients with non-extensive chromoblastomycosis lesions caused by *F. pedrosi* achieved complete recovery following imiquimod treatment, which was linked to an active inflammatory process seen in the skin [29]. While imiquimod can stimulate the immune system, combining it with antifungal medications is recommended for treating large lesions [8].

#### Recent Advances

Recently, it was confirmed that acitretin (20 mg/day), a synthetic retinoid, showed promising potential as a supplementary medication in the treatment of chromoblastomycosis in a single patient with chromoblastomycosis and psoriasis [8]. Combining acitretin, imiquimod, and itraconazole partially controlled the lesions of two patients with persistent chromoblastomycosis lesions, according to a study [30]. It is possible that in these patients, acitretin inhibited skin keratinization, which allowed imiquimod to penetrate the skin and activate immune cells that destroyed intracellular pathogens. As a result, these three medications may work in tandem to treat severe and protracted episodes of chromoblastomycosis [8]. Other compounds that have been investigated (in vitro) in relation to chromoblastomycosis that have fungicidal potential include tricyclazole - an inhibitor of melanin [31], HIV peptidase inhibitors [32], and 1,10-phenanthroline-5,6-dione [33].

Photodynamic therapy (PDT) is also gaining attention as an alternative approach for managing chromoblastomycosis [15]. In a study conducted with ten patients, PDT was able to reduce the size of the lesions by 80–90% after the sixth application [34]. Further research is needed to validate the efficacy of using PDT in treating chromoblastomycosis. However, photodynamic therapy is an expensive treatment option and unfortunately might not be accessible in endemic regions [8].

In 2024, a Global Chromoblastomycosis Working Group was formed by the U.S. Centers for Disease Control and Prevention to promote the scientific and programmatic initiatives for this fungal infection worldwide and included representation from 12 countries [35]. One of the strategies proposed includes developing standardized treatment guidelines that may improve treatment practices. Furthermore, the group suggests developing new antifungals with fewer side effects and drug-drug interactions, longer dosing intervals, researching other triazole antifungals, and assessing combination therapies of itraconazole with other drugs including topical antifungals [35].

### Sporotrichosis

#### Background

Sporotrichosis is a type of subcutaneous mycosis caused by *Sporothrix* species. *S. schenckii*, *S. brasiliensis* and *S. globosa* are the three most common clinically relevant pathogenic species [36]. 95% of sporotrichosis cases are the fixed cutaneous or lymphocutaneous form of sporotrichosis. Other presentations of sporotrichosis can include mucosal, extracutaneous (spread to specific organs) or disseminated cutaneous and disseminated systemic sporotrichosis. Extracutaneous/disseminated cutaneous sporotrichosis can involve infection of joints and bones (osteoarticular), the lungs (pulmonary), or central nervous system (CNS); immunocompromised patients are more likely to have disseminated or systemic sporotrichosis [3, 6, 10, 37–42]. Virulence factors, sporotrichosis species, inoculum of spores, and host immune state all play a role in the clinical manifestations and severity of the disease [10, 43].

Sporotrichosis is found around the world in tropical and subtropical regions. There is a high prevalence in Latin America, Africa and Asia, and different species are present in specific geographic areas: *S. schenckii* in North America and Latin America; *S. brasiliensis* in southeastern South America; *S. globosa* in Asia; *S. schenckii*, *S. globosa* and *S. mexicana* in Australia; and *S. schenckii* and *S. mexicana* in Africa [10, 39, 44, 45]. Zoonotic transmission of *S. brasiliensis* through infected cats has recently been more prevalent, especially in South America, and leads to more severe clinical cases due to its higher virulence compared to other species [6, 10, 39, 43–45].

#### Treatment Landscape

Sporotrichosis rarely resolves on its own and drug treatment is generally required. There is an Infectious Disease Society of America (IDSA) clinical practice guideline; however, this guideline was last updated in 2007 [40, 46, 47]. In the absence of clinical trials and well-controlled studies, the IDSA guideline for treatments, as well as the current drugs and treatment schedules used in clinical practice, are based on case reports, retrospective reviews, and nonrandomized trials [37, 47, 48]. Multiple treatment options for sporotrichosis exist, and the type of medication used depends on the disease severity, species, type of disease presentation, and the patient’s immune status. Identification of *Sporothrix* species and in vitro testing can help determine the course of treatment [10, 40, 48]. Most treatments for cutaneous or lymphocutaneous infections last 3–6 months and are successful in 90% of cases; however, longer treatment of up to 1 year is required for disseminated or systemic cases [10, 45, 48–50].

Ketoconazole was historically used to treat sporotrichosis but is no longer recommended due to its safety profile [3, 46]. Current drug treatments include potassium iodide, itraconazole, terbinafine, amphotericin B, and fluconazole [10, 41, 48]. Potassium iodide (1–4 g/day) has long been used as a first-line treatment but patients have experienced side effects and complications with this regimen [10, 40, 43, 45, 48, 50]. Treatment guidelines recommend itraconazole (100–400 mg/day) as the first choice to treat cutaneous and lymphocutaneous sporotrichosis, which is also preferred by many clinicians [10, 37, 38, 40, 45, 48, 50]. Terbinafine (250–500 mg) is often used as a second-line treatment [9, 10, 37, 40, 43, 45, 48]. Potassium iodide, itraconazole, and terbinafine have occasionally been used in combination to treat sporotrichosis [3, 43].

Amphotericin B, particularly the lipid formulation (3–5 mg/kg/day), is utilized to treat disseminated or severe cases [9, 10, 37–40, 45, 51, 52]. Amphotericin B deoxycholate (0.7–1 mg/kg/day), also known as conventional amphotericin B, is used as an alternative [37, 38, 52]. Conventional amphotericin B is currently the only FDA-approved treatment for sporotrichosis [37, 38, 51–53]. Sequential administration of amphotericin B followed by itraconazole is often used for disseminated or systemic infections, including CNS sporotrichosis [10, 37, 39]. Posaconazole (300–800 mg/day) or fluconazole (150–600 mg/day) have been used as other treatment alternatives, especially when itraconazole is not well tolerated. Fluconazole can penetrate the blood brain barrier better than itraconazole for CNS infections [3, 10, 39, 41, 48, 54–59].

Non-pharmacological options are often used for treating cutaneous sporotrichosis. Cryosurgery can be effective, especially for chronic lesions with limited drug responses or adverse events [60, 61]. Thermotherapy has also been successfully used since the 1960s, particularly for pregnant patients where it is a safer alternative to antifungals [36, 46, 60, 62].

There are still many challenges associated with the treatment of sporotrichosis. Correct diagnosis and identification of species can be difficult as some forms of sporotrichosis mimic other infections. Reliable diagnostics are limited and often unavailable [3, 10, 45]. Current treatments for sporotrichosis vary in efficacy, can have unfavorable pharmacokinetics and pharmacodynamics, and can be toxic [9, 45]. Prolonged use of these drugs may lead to resistance and long-term adverse events including hepatotoxicity, nephrotoxicity, and hypercholesterolemia [3, 9, 10, 38, 47, 48]. There has also been a rise in the number of patients with immunocompromising conditions leading to more presentations of disseminated, systemic, and more severe sporotrichosis infections [36]. Due to these challenges, there is a need for new treatments that can result in clinical cure at a faster rate and that are less toxic [10, 45].

#### Recent Advances

Newer azole agents have been explored for potential treatment of sporotrichosis. Voriconazole and isavuconazole are not recommended as these drugs have high minimum inhibitory concentrations (MICs) in in vitro studies [10, 45]. Ravuconazole has in vitro activity against some *Sporothrix* species, but further research is required [47, 63]. Itraconazole often has side effects, especially with prolonged use; therefore, there have been some recent studies utilizing itraconazole pulse therapy which has shown promising results to provide a safer and cheaper option for administration of the drug [3, 47, 48] There also is potential to utilize pharmaceutical nanotechnology, including solid lipid nanoparticles and nanostructured lipid carriers, which would allow more repurposed drugs to be used in new formulations or dosages, potentially with improved safety and efficacy. Studies with antifungals using these novel formulations have highlighted the potential to overcome drug resistance and toxicity [64].

Other potential treatments may be identified through in vitro studies. In vitro studies demonstrated antifungal effects for a variety of non-azole compounds including pentamidine, miltefosine, and clotrimazole. Recent research has also shown significant antifungal activity for these drugs, as well as crude extracts and natural compounds, in combination with itraconazole. This includes activity against itraconazole-resistant isolates [10].

PDT has been investigated as an alternative therapy for sporotrichosis; however, most PDT studies have been done in vitro or in animals. There have been a few clinical studies, including complete resolution of lesions when treated with topical methylene blue and exposed to sunlight. Further research and studies need to be performed, but the potential to utilize PDT in combination with drug therapy may help reduce toxicity and resistance by limiting time of drug administration [9, 41].

### Mycetoma (Eumycetoma)

#### Background

In 2016, mycetoma was the first fungal disease to be listed as a NTD by WHO) [4]. Mycetoma can be caused by fungi (eumycetoma) or filamentous bacteria (actinomycetoma) and these are clinically indistinguishable [5, 20, 65]. Eumycetoma accounts for about 40% of mycetoma cases worldwide [4]. It is most common in Africa and Southern Asia where it causes considerable morbidity among rural communities [66]; Sudan, Mexico, and India are the countries predominantly affected [4, 5, 20]. The area lying between 15° S and 30° N of the equator is considered the Mycetoma belt [4, 5, 20].

Eumycetoma is a chronic implantation disease characterized by swelling, draining sinuses, and granules in the discharge [5–7, 66]. It begins as a localized skin lesion that, if not recognized early, can affect the skin, fascia, subcutaneous tissues, muscles, bone, and joints [5–7, 66]. There are more than 25 ubiquitous saprotrophic environmental fungi that cause eumycetoma [4]. Four species, *Madurella mycetomatis*, *Trematosphaeria grisea* (*Madurella grisea*), *Scedosporium boydii* (*Pseudoallescheria boydii*), and *Falciformispora senegalensis* (*Leptosphaeria senegalensis*), account for 95% of cases reported globally [4, 5, 65]. The causative agents of eumycetoma were included in the WHO list of fungal priority pathogens in 2022 due to challenges in diagnosis and management [67]. Challenges included the diagnosis of mycetoma, identification of the causative organism, poor responses to antifungal drugs, and a high relapse rate [4, 6, 7, 20, 65]. Eumycetoma does not resolve spontaneously [12].

#### Treatment Landscape

There are no established treatment protocols or guidelines for eumycetoma [4, 12]. Treatment is based on anecdotal experiences, published case reports, and case series [4]. As most drugs used for eumycetoma treatment are unable to penetrate infected tissues adequately to eradicate the causative organism, surgical intervention is often required [4]. Surgery usually involves wide local excision, repeated aggressive debulking and debridement, or amputation in advanced disease [5, 12]. Medical management begins with six months of preoperative antifungals. Antifungal treatment continues postoperatively for at least six months [12] but may be prolonged for years [5, 16, 65]. Eumycetoma is usually refractory to medications [5] and there is a high rate of relapse after surgery [4, 5].

Commonly used drugs for eumycetoma treatment include azoles such as ketoconazole (100–800 mg/day), itraconazole (200–400 mg/day), posaconazole (200 mg/day), voriconazole (400–600 mg/day); amphotericin B (0.5–1.25 mg/kg/day); and terbinafine (500–1000 mg/day); alone or in combination [5, 68]. The causative organisms have different drug susceptibilities [6, 68]. Some respond to voriconazole or itraconazole but they are often resistant to flucytosine and amphotericin B [6].

Oral ketoconazole was the treatment of choice until 2013 when, due to life-threatening hepatotoxicity, the drug was withdrawn from European and Australian markets while FDA imposed “strict relabeling requirements and restrictions on prescription” [69]. Ketoconazole continues to be used in other places, but its popularity has decreased dramatically. Ketoconazole has been replaced by itraconazole as the gold standard for eumycetoma [11, 12, 16, 68]. However, itraconazole has several drawbacks. Cure rates have been reported to be as low as 25.9% and post-operative recurrence rates as high as 27.2%. Treatment duration can stretch to 3 years, reducing compliance and adherence, as well as making patients more vulnerable to adverse effects such as significant drug-drug interactions that raise hepatotoxicity risk. Persistent organism viability in eumycetoma grains and varying bioavailability of itraconazole are also problematic [12]. Moreover, itraconazole can be expensive and challenging to access [67].

Perhaps the most promising antifungal for eumycetoma treatment is fosravuconazole, a prodrug of ravuconazole– a broad-spectrum triazole. In vitro studies of ravuconazole showed good activity against *Madurella mycetomatis*, but it is too expensive to be used for a disease that is almost exclusively prevalent in underprivileged communities [11, 12]. Fosravuconazole was developed as an affordable treatment for Chagas disease and is currently approved in Japan for the treatment of onychomycosis [67]. It has been recently assessed in the first double-blind randomized clinical trial in eumycetoma patients at the Mycetoma Research Center (MRC) in Sudan (NCT03036226) in collaboration with the Drugs for Neglected Diseases initiative (DNDi) and Eisai [67]. Fosravuconazole was not shown to be superior to itraconazole, but the efficacy rates were comparable. However, it had a better safety profile and less drug-drug interactions than itraconazole and required only weekly administrations compared to twice daily dosing for itraconazole. This has the potential to increase adherence leading to better outcomes in the real world [67].

Voriconazole (400–600 mg/day) and posaconazole (800 mg/day) are other azole alternatives [12, 16] that have shown in vitro activity and are reported to have favorable clinical outcomes in eumycetoma [7, 11, 12]. Terbinafine (1 g/day) has also been used successfully for mycetoma, particularly for *Madurella mycetomatis* in combination with surgery [12, 16]. Terbinafine can be used as an alternative to itraconazole when it is unavailable, or they can be combined in refractory cases [16]. Amphotericin B has also been used to treat eumycetoma. However, its use has been limited by toxicity and the need for hospitalization. Several cases where liposomal amphotericin B (1–3 mg/kg/day) was used did not show satisfactory clinical responses and some patients experienced severe adverse events [12].

Despite the promise, all these antifungals present similar challenges that have plagued the treatment of mycetoma. The long treatment duration has made it hard to evaluate the slow improvement over months of therapy [6] while treatment failure and relapses have been reported [7, 12]. The high cost, hepatotoxicity, and the relative lack of studies regarding their response have also limited their use [11, 12, 68].

#### Recent Advances

Drug repurposing continues to be the most promising avenue for drug discovery for eumycetoma [12] and employing new strategies can improve efficacy of the currently used medications. More studies on antifungal susceptibility, especially for fosravuconazole and voriconazole, and in vivo animal model studies with larger sets of well-identified isolates could optimize antifungal choice and improve patient outcomes [7, 16]. Given the promise of fosravuconazole, further research is needed to assess effectiveness and cure rates with shorter durations and without surgery [67]. Itraconazole’s variable and unsatisfactory clinical efficacy could be partially due to its poor pharmacokinetic profile. Maintaining effective and safe serum levels could be achieved via therapeutic drug monitoring techniques. Pharmaceutical technologies have also been developed to enhance oral bioavailability. For instance, super bioavailable (SUBA) itraconazole exhibits reduced pharmacokinetic variability, fewer adverse events, and effectiveness than conventional itraconazole at lower doses [70, 71]. Adopting these technologies could enhance therapeutic efficacy while reducing treatment cost [12].

Amphotericin B has historically performed poorly in the treatment of mycetoma, but some innovations have shown promise. Amphotericin B impregnated absorbable calcium sulfate beads (Stimulan) applied into bony defects after surgery and intralesional administration of amphotericin B have shown good clinical outcomes with fewer side effects [11], but these are new treatments and are expensive. There is also a concern that diffusion of the drug into lesions may not be possible with the multilobulated lesions commonly seen with *Madurella mycetomatis*. This is also a painful procedure and may cause dissemination of the infection [12].

The addition of non-steroidal anti-inflammatory drugs or oral prednisolone have been reported to improve clinical outcomes for eumycetoma [16] which warrants further investigation. Other drugs such as natamycin, eficonazole, and amorolfine have also been tested (in vitro) against some isolates of the genus causing eumycetoma but high MICs were reported [7].

Besides medical intervention, one study reported public health initiatives such as using local care, providing health education, training to detect and treat early, improving hygiene, and removing environmental sources of infection reduced amputation rates from 63 to 12% in an endemic village in Sudan [72]. This model of care could be replicated in other endemic areas [72].

Although *de novo* drug discovery has many challenges, organizations such as the Drugs for Neglected Diseases *initiative* (DND*i*) are supporting discovery for neglected diseases through projects such as the Mycetoma Open Source project. This open-access database that is publicly driven, aims to discover new drugs and to optimize available leads for the management of eumycetoma [12]. Such endeavors that lower the cost of research and development for pharmaceutical companies can lead to more drug discovery for mycetoma in the future.

## Conclusion

The current treatment landscape of FDA-approved drugs for fungal NTDs is extremely limited, consisting only of amphotericin B for sporotrichosis and ketoconazole for chromoblastomycosis, though the latter is no longer used in clinical practice. Researchers and clinicians continue to explore treatment options and combinations, but the diseases remain difficult to treat, often requiring prolonged therapy and, in some cases, surgical interventions. Even so, cure rates are low, and recurrence is common.

As a result, clinicians treating patients with fungal NTDs routinely use drugs off-label to treat their patients’ infections. Medications that are frequently repurposed to treat these diseases include itraconazole, posaconazole, voriconazole, fosravuconazole, amphotericin B, terbinafine, potassium iodide, and 5-flucytosine. These antifungal agents may also be used sequentially or in combination with one another. Occasionally, other agents such as immunomodulators (e.g., imiquimod), steroids (e.g., prednisolone), or non-steroidal anti-inflammatory agents are used in combination with more traditional antifungal agents.

There is, however, significant toxicity associated with these antifungals. Expense and inadequate availability of drugs remain additional challenges. Further development of less toxic or faster acting medications may help treat these conditions. This may depend on refinement of existing repurposed drugs (e.g., formulation changes, nanotechnology).

For fungal NTDs, the limited approved treatments benefit relatively few patients, and drugs are routinely used off-label. Until recently, this clinical experience was rarely captured and efforts to learn from real-world use of existing drugs was limited to occasional publications in peer-reviewed journals. CURE ID, an FDA, and National Center for Advancing Translational Sciences of the National Institutes of Health (NCATS/NIH) initiative, is a public, electronic treatment registry to capture case reports of off-label use by clinicians for diseases with unmet medical needs. It was established to address this gap and to enable the clinical community to learn from real-world treatment experiences. CURE ID systematically collects data on the off-label use of drugs for difficult to treat conditions through an electronic case reporting tool. Clinicians who are facing these challenging conditions are encouraged to share their treatment experiences on CURE ID (https://cure.ncats.io). Data regarding off-label use could help generate hypotheses about the potential effectiveness of certain drugs. These findings then need to be investigated in randomized controlled trials to verify or refute the observational findings. Supporting large, randomized controlled trials of repurposed drugs is likely the most efficient way to produce the high-quality evidence needed by clinicians to make treatment decisions for their patients with fungal NTDs.

## Supplementary Material

Supplementary Material 1 (Search Strategy)

**Supplementary Information** The online version contains supplementary material available at https://doi.org/10.1007/s12281-025-00504-z.

## Data Availability

No datasets were generated or analysed during the current study.
