# Supplementary material for: The Current Landscape of Repurposed Drugs for Fungal Neglected Tropical Diseases
Source: Curr Fungal Infect Rep. Author manuscript; Available in PMC 2025 Aug 22. (PMC12363401; doi:10.1007/s12281-025-00504-z)
Supplement: Supplementary Material 1 (Search Strategy) [file NIHMS2092398-supplement-Supplementary_Material_1__Search_Strategy_.docx]

**Supplementary Material**

The search strategy used to extract manuscripts from PubMed was:

("Mycetoma"[Mesh] OR "Chromoblastomycosis"[Mesh] OR "Sporotrichosis"[Mesh] OR "sporotrich*"[tw] OR "eumycetom*"[tw] OR "implantation mycos*"[tw] OR "subcutaneous mycos*"[tw]) AND ("Drug therapy"[Mesh] OR "Therap*"[tw] OR "Treatment*"[tw] OR "Treatment*"[tw] OR "Chemotherap*"[tw] OR "pharmacotherapy*"[tw] OR "Drug therap*"[tw] OR "Therapeutics"[Mesh] OR "therapy" [subheading] OR "Amputation, surgical"[Mesh]) Filters: Adaptive Clinical Trial, Books and Documents, Classical Article, Clinical Study, Clinical Trial, Clinical Trial, Phase I, Clinical Trial, Phase II, Clinical Trial, Phase III, Clinical Trial, Phase IV, Comparative Study, Controlled Clinical Trial, Editorial, Equivalence Trial, Evaluation Study, Guideline, Meta-Analysis, Multicenter Study, Observational Study, Practice Guideline, Pragmatic Clinical Trial, Randomized Controlled Trial, Review, Systematic Review, Technical Report, Validation Study, English, Humans, from 2019/01/01 - 2024/11/02.
